# Bacteriologic and plasmid analysis of etiologic agents of conjunctivitis in Lagos, Nigeria

**DOI:** 10.1007/s12348-011-0024-z

**Published:** 2011-04-05

**Authors:** Bamidele Abiodun Iwalokun, Afolabi Oluwadun, Kehinde Adewale Akinsinde, Mary Theressa Niemogha, Fransisca Obiageri Nwaokorie

**Affiliations:** 1 Department of Biochemistry Nutrition, Nigerian Institute of Medical Research (NIMR), Yaba, Lagos, Nigeria; 2 Department of Medical Microbiology and Parasitology, Olabisi Onabanjo University, Sagamu, Ogun State Nigeria; 3 Department of Molecular Biology and Biotechnology, Nigerian Institute of Medical Research (NIMR), Yaba, Lagos, Nigeria; 4 Department of Medical Microbiology and Parasitology, University of Benin, Benin, Edo State Nigeria

**Keywords:** Conjunctivitis, Bacterial pathogens, Lagos

## Abstract

**Background:**

Conjunctivitis, an inflammation of the conjunctiva, is one of the most common eye problems affecting all age groups in Nigeria. A better understanding of its epidemiology and the antibiotic susceptibility of etiologic bacterial agents is crucial for the initiation of preventive and therapeutic measures. This study determined the distribution and patterns of bacterial infections in Nigerian patients with conjunctivitis. Antibiotic resistance patterns and the plasmid profiles of these pathogens were also investigated.

**Methodology:**

A total of 83 consecutive and non-duplicate conjunctival specimens were collected from patients attending eye clinics at three different hospitals in Lagos, Nigeria, between February and September 2010. Specimens were cultured on standard bacteriologic media and the recovered isolates speciated using standard techniques. Susceptibility of pathogens to antibiotics and plasmid DNA extraction were carried out by disk diffusion and alkaline lysis methods. Conjugation experiment was done with rifampicin-resistant *Escherichia coli* DH5α as the recipient cell. Data were analyzed using the chi-square test.

**Results:**

All the specimens were culture-positive, yielding a total of 155 bacterial isolates. Gram-positive cocci comprising *Staphylococcus aureus* (27.7%) and coagulase-negative *Staphylococcus* sp. (22.6%) accounted for 50.3% (78 of 155) of conjunctivitis cases, followed by Gram-positive bacilli (22.6%), Gram-negative bacilli (21.3%), and Gram-negative cocci (4.5%). *Corynebacterium* spp. were the most commonly isolated Gram-positive bacilli accounting for 16.1% of conjunctivitis cases. *Pseudomonas aeruginosa* topped with 9.7% as the most commonly isolated Gram-negative bacilli. Other Gram-negative bacilli in order of their isolations were *E. coli* (6.5%), *Proteus* sp. (3.2%), *Klebsiella* sp. (1.9%), and *Enterobacter aerogenes* (1.9%). *Moraxella* spp. were the only Gram-negative cocci isolated, and they accounted for 4.5% of the total conjunctival infections. Further analysis of the complexity of infections showed that 25 specimens elicited mono-infections, while cases of polymicrobial infections caused by two pathogens and three or more pathogens constituted 51.8% and 18.1% of conjunctivitis specimens screened, respectively. The disparity in the percentage contribution of three infection patterns was significant (*P* < 0.05). Antibiotic susceptibility testing revealed chloramphenicol and ofloxacin as the least and most active antibiotics tested as 99 (63.9%) and 149 (96.1%) of the 155 recovered isolates were sensitive to them. On the whole, the least susceptible pathogen was *P. aeruginosa* with sensitivities ranging from 20% to 80%, while *Moraxella* sp. represented the most sensitive pathogen with sensitivities ranging from 71.4% to 100%. Other bacterial isolates also elicited antibiotic sensitivities in the range of 33.3–100%. A total of 101 isolates were screened for plasmids, of which 45 harbored plasmids, yielding a plasmid frequency of 44.6%. Conjugal transfer of resistance to chloramphenicol, ampicillin, and streptomycin was detected in the transconjugants after the mating experiment. The antibiotic resistances were transferred either singly or in combination from six of the seven selected donor strains. The antibiotic resistance pattern transferred by these donor strains was partial and was associated with the transfer of R plasmids of sizes 21.3, 15.2, and 5.0 kb from three of the six transferable strains. The frequencies of transfer of antibiotype or R plasmids to the transconjugants ranged from 1.8 × 10^−7^ to 1.4 × 10^−5^ transconjugants per donor strain.

**Conclusion:**

Conjunctivitis as an eye problem in Lagos is polymicrobial with infections associated with transferable R plasmids for chloramphenicol, ampicillin, and streptomycin. Continuous surveillance of conjunctivitis in relation to etiology, drug susceptibility, and plasmid transferability in the study area is therefore recommended.

## Introduction

Both Gram-positive and Gram-negative bacteria are increasingly becoming important both clinically and therapeutically as biological agents of ocular infections throughout the world. Various forms of ocular infections caused by pathogenic bacteria have been reported by different investigators. They include conjunctivitis, cornea perforation, orbital cellulitis, endophthalmitis, panophthalmitis and dacryocystitis, internal and external hordeolum, keratitis, scleritis, and canaliculitis [1–3]. Some of these infections carry poor prognosis as patients are at risk of losing either their sights or life, or both [2, 4]. This has necessitated the prompt detection of the etiologic agent and the timely institution of appropriate antibiotic treatment for patients with ocular infections. In Nigeria, conjunctivitis is one of the most common eye problems that affect all age groups. An epidemic of conjunctivitis was reported in Nigeria in 1971 [5]. In neonates, the incidence of conjunctivitis has been reported to be 18 per 1,000 live births, and predisposing factors have been found to include vaginal delivery, asphyxia neonatorum, and prolonged rupture of the membrane [6]. A previous epidemiological study carried out by Abiose et al. [7] revealed conjunctivitis as among the causes of ophthalmic defects needing urgent medical attention in 10.4% of the 5,220 post-primary school Nigerian children screened. The routes of transmission of conjunctivitis have been identified to include air droplets, use of contaminated water for eye wash, hand to eye contact, and endogenous sources [2]. The pathology of conjunctivitis is symptomized by redness of the eye, grittiness, photophobia, and watery discharge. There may also be cornea involvement leading to subepithelial and epithelial keratitis. If untreated, conjunctivitis may degenerate pathologically to sight and life-threatening complications [8, 9]. Therefore, the early identification of etiologic agents and prompt institution of appropriate antibiotic therapy are essential for optimal eye care and restoration of a good eye health. Studies by investigators from other countries indicate that Gram-positive and Gram-negative bacteria are the most commonly isolated pathogens in patients with conjunctivitis, but variations exist in aetiologies, drug susceptibilities of pathogens, and antibiotic resistance mechanisms. Gram-positive bacteria such as *Staphylococcus aureus*, non-coagulase-positive *Staphylococci*, *Bacillus* sp., *Corynebacterium* sp., *Streptococcus pneumoniae*, *Streptococcus pyogenes*, and *Streptococcus viridans* have been implicated as aetiologies of conjunctivitis in patients [8, 10, 11]. In Gram-negative-mediated conjunctivitis, pathogens such as *Pseudomonas aeruginosa*, *Escherichia coli*, *Proteus* sp., *Moraxella* sp., and *Neisseria gonorrhoeae* have been isolated from conjunctival samples as etiologic agents [8, 12]. There are also indications that these pathogens elicit dynamism in order to achieve clonal success as agents of conjunctivitis coupled with their increasing propensity to develop resistance against the commonly used antibiotics in the form of eye drops and ointment to treat conjunctivitis. Plasmids, which are extrachromosomal double-stranded DNA materials, have been found to be useful for pathogens’ genetic diversity and prowess as infectious agents. Profiling pathogens for their harbored plasmids has been found to be very useful in epidemiological studies, diagnosis, and elucidation of mechanisms of drug resistance [13]. Plasmids have also been found useful in knowing whether two or more strains of a pathogen evolve from the same microorganism, thereby providing a reliable insight into the genetic relatedness of pathogens in an environment [14, 15].

In Nigeria, there have not been adequate data regarding etiologic agents of conjunctivitis coupled with lack of updates on trends in antibiotic resistance patterns of ocular pathogens to inform treatment guidelines in the care of eye-infected patients. This is proposed to play a role in the deteriorating eye health of patients despite the use of antibiotics.

This study was conducted to determine antibacterial susceptibility and characterize the plasmids harbored by bacteriologic agents of conjunctivitis in Lagos, Nigeria.

## Materials and methods

### Specimen and bacteria identification

A total of 83 consecutive and non-duplicate conjunctival specimens were collected from patients attending eye clinics at Lagos State University Teaching Hospital and two district hospitals in Lagos, Nigeria, between February and September 2010. The patients were clinically diagnosed to have conjunctivitis through history taking and clinical examination of the eye and were enrolled into the study after obtaining an informed consent.

Specimens were collected by swiping a broth-moistened swab across the conjunctiva of the affected eye(s) per patient. Specimens were transported in cold boxes within 4 h of collection. To grow bacteria, specimens were inoculated directly onto sheep blood agar, chocolate agar, MacConkey agar, and brain heart infusion broth (BHI). Colonies obtained in the primary plates and BHI cultures were further subcultured in the various solid growth media including mannitol salt agar. Direct microscopic examinations such as 10% KOH wet mounting and gram staining were carried out, and each bacterial isolate was speciated via standard biochemical tests [16].

### Antibiotic susceptibility testing

In vitro susceptibility to antibiotics of the bacterial isolates and transconjugants was done by the Kirby–Bauer disk diffusion method [17] on Muller–Hinton (MH) agar. An inoculum of each bacterial isolate was prepared by diluting its overnight culture to 0.5 MacFarland standard (1.5 × 10^8^ cfu/mL) using sterile MH broth. The standard antibiotic disks used from Oxoid (UK) were chloramphenicol 30 μg, gentamicin 30 μg, ampicillin 30 μg, ofloxacin 5 μg, amoxicillin 30 μg, nitrofurantoin 200 μg, amikacin 25 μg, and streptomycin 30 μg. The results obtained were interpreted as sensitive or resistant according to the National Committee on Clinical Laboratory guidelines [18]. *E. coli* ATCC 25922 was used as a quality control strain.

### Conjugation experiment and plasmid isolation

Conjugation experiment was carried out according to Willets [19] using rifampicin-resistant *E. coli* DH5α as the recipient cell and seven selected representatives of Gram-negative (*n* = 5) and Gram-positive bacterial cells (*n* = 2) as donor strains. The initial donor-to-recipient ratio of 1:20 was used for mating. Transconjugants were selected on MH agar plate containing 300 μg/mL of rifampicin and 50 μg/mL of ampicillin or chloramphenicol. Plasmid extraction of donor strains and transconjugants was done using the alkaline lysis method of Takahashi and Nagano [20]. Plasmid DNA bands were detected by electrophoresis on 0.8% horizontal agarose gel pre-stained with ethidium bromide (0.5 μg/mL) and visualized under UV light. The sizes of the plasmid DNA bands were determined by extrapolation based on the mobilities of HindIII digested λ DNA co-electrophoresed with the plasmid DNA samples [21]. Antibiotic susceptibility assay was also carried out on the transconjugants.

### Statistical analysis

Data on the complexity of infections, expressed as percentage, were analyzed using the chi-square (*χ* ^2^) test. A value of *P* <0.05 was considered to be significant.

## Results

**Fig. 1:**
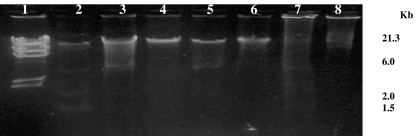
Agarose gel electrophoresis of ocular pathogens recovered as mono-infections. *Lane 1* HindIII λ DNA markers; *Lane 2 E. coli*; *Lanes 3–5 P. aeruginosa*; *Lane 6 E. aerogenes*; *Lane 7 K. pneumoniae*; *Lane 8 Moraxella* sp.

**Fig. 2:**
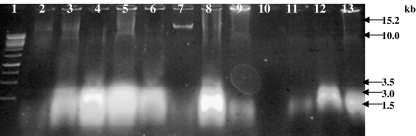
Agarose gel electrophoresis of ocular pathogens recovered as polymicrobial infections. *Lane 1* 10-kb DNA marker; *Lanes 2 and 3 S. aureus*; *Lane 4 Bacillus* sp.; *Lane 5 Corynebacterium* sp.; *Lane 6 Bacillus* sp.; *Lane 7 Moraxella* sp.; *Lane 8 P. aeruginosa*; *Lane 9 Klebsiella* sp.; *Lane 10* negative control (sterile water); *Lanes 11–13* Coagulase-negative *Staphylococcus* sp.

Over a period of 3 months, 83 consecutive and non-duplicated conjunctival specimens of patients suspected to have conjunctivitis were collected at the eye clinics of Lagos General Hospital and two district hospitals in Lagos. The profiles of Gram-positive and Gram-negative bacteria recovered from processed samples of patients suspected to be having conjunctivitis are summarized in Table 1. All the specimens were culture-positive, yielding a total of 155 bacterial isolates. Gram-positive cocci comprising *S. aureus* (27.7%) and coagulase-negative *Staphylococcus* sp. (22.6%) accounted for 50.3% (78 of 155) of conjunctivitis cases, followed by Gram-positive bacilli (22.6%), Gram-negative bacilli (21.3%), and Gram-negative cocci (4.5%). *Corynebacterium* spp. were the most commonly isolated Gram-positive bacilli, accounting for 16.1% of conjunctivitis cases. *P. aeruginosa* topped with 9.7% as the most common Gram-negative bacillus. Other Gram-negative bacilli in order of their isolation rates were *E. coli* (6.5%), *Proteus* sp., (3.2%), *Klebsiella* sp. (1.9%), and *Enterobacter aerogenes* (1.9%). *Moraxella* spp. were the only Gram-negative cocci isolated, and they accounted for 4.5% of the total conjunctival infections. Further analysis of complexity of infections showed that 25 specimens elicited mono-infections (Fig. 1), while cases of polymicrobial infections caused by two pathogens and three or more pathogens constituted 51.8% and 18.1% of conjunctivitis specimens screened, respectively (Fig. 2). The disparity in the percentage contribution of three infection patterns was significant (*χ* ^2^ = 21.8, *P* = 1.8 × 10^−5^; Table 1).

**Table 1:** Gram-positive and Gram-negative bacteria recovered from the eyes of patients with conjunctivitis

Organism	No. (%) recovered	Complexity of infections			*χ* ^2^	*P* value
		Mono-infection	Polymicrobial infection no. (%)			
		No. (%)	Two organisms	More than two organisms		
Total Gram-positive cocci	78 (50.3)	25 (30.1)	43 (51.8)	15 (18.1)	21.8	1.8E−5
*S. aureus*	43 (27.7)					
Coagulase-negative *Staph*	35 (22.6)					
Total Gram-positive bacilli	35 (22.6)					
*Bacillus* sp.	10 (6.5)					
*Corynebacterium* sp.	25 (16.1)					
Total Gram-negative bacilli	33 (21.3)					
*P. aeruginosa*	15 (9.7)					
*E. coli*	10 (6.5)					
*Proteus* sp.	5 (3.2)					
*Enterobacter aerogenes*	3 (1.9)					
*Klebsiella* sp.	3 (1.9)					
Gram-negative cocci	7 (4.5)					
*Moraxella* sp.	7 (4.5)					
Total	155 (100)

In vitro susceptibilities of the recovered Gram-positive and Gram-negative bacterial isolates to nine antibiotics are shown in Table 2. Chloramphenicol and ofloxacin were found to be the least and most active antibiotics tested as 99 (63.9%) and 149 (96.1%) of the 155 recovered isolates were sensitive to them. On the whole, the least susceptible pathogen was *P. aeruginosa* with sensitivities ranging from 20% to 80%, while *Moraxella* sp. represented the most sensitive pathogen with sensitivities ranging from 71.4% to 100%. Other bacterial isolates also elicited antibiotic sensitivities in the range of 33.3–100%.

**Table 2:** In vitro antibiotic susceptibilities of bacterial isolates recovered from conjunctival specimens of patients with suspected conjunctivitis

Organism	No. tested	No. (%) of susceptible bacterial isolates to antibiotics tested								
		CHL	CTX	AMK	GEN	STR	CAZ	AMP	AMO	OFL
Total Gram-positive cocci	78	51 (65.3)	71 (91)	69 (88.5)	64 (82.1)	61 (78.2)	73 (93.6)	62 (79.5)	67 (85.9)	78 (100)
*S. aureus*	43	24 (55.8)	38 (88.4)	37 (86)	34 (79.1)	29 (67.4)	38 (88.4)	33 (76.7)	35 (81.4)	43 (100)
Coagulase-negative *Staph*	35	27 (77.1)	33 (94.3)	32 (91.4)	30 (85.7)	32 (91.4)	35 (100)	29 (82.9)	32 (91.4)	35 (100)
Total Gram-positive bacilli	35	28 (80)	31 (88.6)	33 (94.3)	33 (94.3)	30 (85.7)	34 (97.1)	31 (88.6)	33 (94.3)	35 (100)
*Bacillus* sp.	10	9 (90)	10 (100)	8 (80)	9 (90)	8 (80)	10 (100)	8 (80)	10 (100)	10 (100)
*Corynebacterium* sp.	25	19 (76)	21 (84)	25 (100)	24 (96)	22 (88)	24 (96)	23 (92)	23 (92)	25 (100)
Total Gram-negative bacilli	36	15 (41.7)	29 (80.6)	30 (83.3)	28 (77.8)	24 (66.7)	30 (83.3)	23 (63.9)	27 (75)	29 (80.6)
*P. aeruginosa*	15	3 (20)	12 (80)	11 (73.3)	10 (66.7)	8 (53.3)	12 (80)	9 (60)	11 (73.3)	9 (60)
*E. coli*	10	6 (60)	8 (80)	8 (80)	8 (80)	8 (80)	9 (90)	7 (70)	8 (80)	9 (90)
*Proteus* sp.	5	3 (60)	5 (100)	5 (100)	4 (80)	4 (80)	4 (80)	4 (80)	4 (80)	5 (100)
*Enterobacter aerogenes*	3	2 (66.7)	3 (100)	3 (100)	3 (100)	3 (100)	3 (100)	2 (66.7)	3 (100)	3 (100)
*Klebsiella* sp.	3	1 (33.3)	1 (33.3)	3 (100)	3 (100)	1 (33.3)	2 (66.7)	1 (33.3)	1 (33.3)	3 (100)
Total Gram-negative cocci	7	5 (71.4)	7 (100)	7 (100)	7 (100)	5 (71.4)	7 (100)	5 (71.4)	7 (100)	7 (100)
*Moraxella* sp.	7	5 (71.4)	7 (100)	7 (100)	7 (100)	5 (71.4)	7 (100)	5 (71.4)	7 (100)	7 (100)
Total	155	99 (63.9)	138 (89)	139 (89.7)	132 (85.2)	120 (77.4)	144 (92.9)	121 (78.1)	134 (86.5)	149 (96.1)

*CHL* chloramphenicol, *CTX* cefotaxime, *CAZ* ceftazidime, *AMK* amikacin, *STR* streptomycin, *AMP* ampicillin, *AMO* amoxicillin, *OFL* ofloxacin, *GEN* gentamicin

A total of 101 isolates were screened for plasmids, of which 45 harbored plasmids, yielding a plasmid frequency of 44.6%.

Tables 3 and 4 depict outcomes of the conjugation experiment. Conjugal transfer of resistance to chloramphenicol, ampicillin, and streptomycin was detected in the transconjugants after the mating experiment. The antibiotic resistances were transferred either singly or in combination from six of the seven selected donor strains. The antibiotic resistance pattern transferred by these donor strains was partial and was associated with the transfer of R plasmids of sizes 21.3, 15.2, and 5.0 kb from three of the six transferable strains (Tables 3 and 4). The frequencies of transfer of antibiotype or R plasmids to the transconjugants ranged from 1.8 × 10^−7^ to 1.4 × 10^−5^ transconjugants per donor strain.

**Table 3:** Percentage of bacterial isolates harboring plasmids from the conjunctival specimens

Organism	No. tested	No. (%) of bacterial isolates	
		Plasmid-positive	Plasmid-negative
Total Gram-positive cocci	50	19 (38)	31 (72)
*S. aureus*	25	11 (44)	14 (56)
Coagulase-negative *Staph*	25	8 (32)	17 (68)
Total Gram-positive bacilli	18	7 (38.9)	11 (61.1)
*Bacillus* sp.	8	4 (50)	4 (50)
*Corynebacterium* sp.	10	3 (30)	7 (70)
Total Gram-negative bacilli	26	18 (69.2)	8 (30.9)
*P. aeruginosa*	10	5 (50)	5 (50)
*E. coli*	7	6 (85.7)	1 (14.3)
*Proteus* sp.	3	2 (66.7)	1 (33.3)
*Enterobacter aerogenes*	3	2 (66.7)	1 (33.3)
*Klebsiella* sp.	3	3 (100)	0 (0)
Total Gram-negative cocci	7	1 (14.3)	6 (85.7)
*Moraxella* sp.	7	1 (14.3)	6 (85.7)
Total	101	45 (44.6)	56 (55.4)

**Table 4:** Conjugal transfer of antibiotic resistance and plasmids to *E. coli* DH5α by some of the selected bacterial isolates recovered from patients with conjunctivitis

Donor strain	Antibiotype	Plasmid profile	Antibiotype transferred to *E. coli* DH5α	R plasmid transferred to *E. coli* DH5α	Conjugation frequency^a^
*P. aeruginosa* NG-02	Chl Str Amp Gen	21.3, 5.0, 1.2, 1.0	Chl Str Amp	21.3, 5.0	1.8 × 10^–7^
*P. aeruginosa* NG-07	Amo Amp Ctx Ofl	21.3	Amp	–	1.4 × 10^–5^
*E. coli* NG-05	Chl Amp Str Amk	21.3, 5.0	Amp Str	21.3	1.9 × 10^–7^
*E. coli* NG-01	Chl Amp Gen Amo	15.2, 10.0, 4.1	Chl Amp	15.2	1.8 × 10^–6^
*Klebsiella oxytoca* NG-03	Chl Str	4.1, 2.7	Chl	–	2.1 × 10^–7^
*S. aureus* NG-18	Amp Str Amo	15.2, 9.0, 5.0, 2.0	Str	–	1.9 × 10^–5^
*S. epidermidis* NG-22	Chl Amp Str	15.2, 2.0	–	–	ND

*ND* not determined, *Chl* chloramphenicol, *Amp* ampicillin, *Str* streptomycin, *Amo* amoxicillin, *Gen* gentamicin, *Amk* amikacin, *Ofl* ofloxacin, *Ctx* cefotaxime

^a^Conjugation frequency is defined as the number of transconjugants as a proportion of total bacterial count in the nonselective medium

## Discussion

The infections of the eye including conjunctivitis are a major cause of hospital consultation in Nigeria and are associated with hospitalization and mortality rates of 0.3–3% [22–24]. The present study shows that conjunctivitis in Lagos is caused by multiple bacterial aetiologies involving both Gram-positive and Gram-negative pathogens with varying antibiotic susceptibility patterns and plasmid carriage ability. The transfer of antibiotic resistance and R plasmids by some of the recovered strains to *E. coli* was also revealed. The observed etiologic pattern of Gram-positive pathogens predominated by *S. aureus* (27.7%) and non-coagulase *Staphylococci* (22.6%) as causative agents of conjunctivitis in this study is similar to previous reports by Iroha et al. [6] who reported 37.4% and 12.3% as the isolation rates for *S. aureus* and coagulase-negative *Staphylococci* in the same environment. In southeast Nigeria, Ubani [25] also recovered *S. aureus* (23.7%) and *Staphylococcus albus* (19.3%) as the predominant Gram-positive pathogens of ocular infections in patients. In other countries of the world such as India, isolation rates of 25% and 18.3% have been reported in *S. aureus* and non-coagulase *Staphylococci* as ocular pathogens, respectively [8]. Generally, the predominance of *Staphylococci*, *Bacillus* sp., and *Corynebacterium* sp. as major ocular pathogens might be due to the fact that these organisms represent the major flora of the eye lid and the conjunctiva and under normal conditions, their clinical manifestations are averted by eye innate immune defense system constituted by tear flow, secretory immunoglobulin, and the presence of cidal agents such as lysozyme and lactoferrin [9, 26]. Other Gram-positive pathogens such as *S. pneumoniae*, *S. pyogenes*, *S. viridans*, and *Haemophilus influenzae* were not found in this study and were not reported by Iroha et al. [6]. This suggests consistency in the Gram-positive etiology of conjunctivitis in Lagos, even though non-coagulase *Staphylococci* are now trending toward equality with *S. aureus* in causing conjunctivitis in the study area. Contrastingly, these pathogens were implicated as ocular pathogens in southeast Nigeria [25], India [8], Hong Kong [27], USA [12], Singapore [9], and Ghana [10], reconfirming previous reports that ocular pathogens vary in etiology in different countries and different locations within a country [8, 25, 28]. Our non-recovery of other Gram-positive ocular pathogens may also be due to the focus of this study, which centered on conjunctivitis compared with other ocular infections included in the Indian and southeastern Nigerian studies [8, 25]. With regards to Gram-negative pathogens in ocular infections in Lagos, we found *P. aeruginosa* as the predominating strain with an isolation rate of 9.7%, whereas in a previous study in the same environment, *Klebsiella pneumoniae* was the most commonly isolated pathogen (12.9%) followed by *P. aeruginosa* (8.2%). Therefore, our finding indicates a changing trend in the Gram-negative etiology of conjunctivitis in Lagos. In other parts of the country, *K. pneumoniae* was also most commonly isolated, followed by *E. coli* coupled with the involvement of other Gram-negative pathogens such as *N. gonorrhoeae* and *Neisseria meningitides*. This again corroborates the influence of locations on the etiology of ocular infections. Unlike the present study, which is similar in context to the study in southeast Nigeria, the previous Lagos study focused on neonates who have predilections to infections due to *E. coli* and *Klebsiella* sp. in that age group. In the present study, the 83 conjunctival samples tested were obtained from patients of all age groups, with 10.8% being children aged 2–5 years, 27.7% were aged 6–13 years representing older children, 21.7% were adolescents aged 14–19 years, and 39.8% were adults.

Our results of pathogens’ antibiotic susceptibilities suggest that in vitro, chloramphenicol has deteriorated as an anti-conjunctival agent with an overall efficacy of 63.9% and being 55.8–90% and 20–71.4% effective against Gram-positive and Gram-negative bacteria isolated, respectively. In an increasing order anti-conjunctival activity, other tested antibiotics’ activities are streptomycin < ampicillin < gentamicin < amoxicillin < cefotaxime < amikacin < ceftazidime < ofloxacin, with efficacy ranging from 77.4% to 96.1%. In clinical context, our findings connote a possible reduction in the therapeutic relevance of chloramphenicol and gentamicin in the treatment of conjunctivitis in Lagos as these antibiotics in the form of eye drops are most commonly available and used in the study area. Given the higher anti-conjunctival efficacies of amikacin, cefotaxime, and ofloxacin, the availability as eye preparations and the empirical use of these antibiotics are strongly recommended in the management of conjunctivitis in Lagos. Elsewhere, fluoroquinolones such as ofloxacin and gatifloxacin are also the current recommended drugs of choice in the treatment of ocular infections [8–10, 29, 30]. However, to prevent and control the development of antibiotic resistance by ocular pathogens to these antibiotics and even optimize the therapeutic applications of chloramphenicol and gentamicin, institution of therapy based on the antibiogram profile of pathogens is highly essential. Furthermore, our observation that 69.9% of conjunctivitis cases screened are polymicrobial in etiology provides an indication for a complex epidemiological situation of conjunctivitis in this environment. Therefore, institution of appropriate anti-conjunctival therapy in afflicted patients is very important as this would avert further complications and improve the prognosis of infection. Based on our findings, such appropriateness in medication may warrant the use of two or more antibiotics especially for cases in which both Gram-positive and Gram-negative pathogens are involved.

The present study has also documented the involvement of plasmids as factors responsible for antibiotic resistance in some of the recovered pathogens since these resistances were partly transferred to *E. coli* DH5α by conjugation. Therefore, our findings suggest the emergence and active transfer of antibiotic resistance and R plasmids among the circulating strains causing conjunctivitis in Lagos. In this study, antibiotics such as chloramphenicol, streptomycin, and ampicillin were easily transferred from a multidrug-resistant ocular pathogen to *E. coli*. The co-transfer of plasmids of sizes 21.5, 15.2, and 5.0 kb suggests that these extrachromosomal DNAs are R plasmids, even though curing experiment was not done. The minimum size of a plasmid with an efficient conjugation system has been reported to be >15 kb [14]. Therefore, the presence of the 5.0-kb plasmids in the transconjugants implies that the larger molecular size plasmids (i.e., 21.3 and 15.2 kb) might serve as vehicles for the transfer of lower molecular weight plasmids such as 5.0 kb to *E. coli* DH5α. This also implies that the mechanism of plasmid mobilization among the ocular pathogens may entail the use of larger sized plasmids as vehicles in addition to the classical transfer systems such as conjugation that require considerable genetic information, as previously reported by Smith and Linggood [31], Achmith and Helmuth [32], Christiansen et al. [33], and Jamieson and Bremner [14]. The result from the conjugation experiment also revealed that the varied drug-resistant exconjugants arose at a frequency range of 10^−5^–10^−7^ per donor cell. These evolution rates are similar to those of previously reported epidemiological and clinically important pathogens in Nigeria, and some of these pathogens were also recovered in this study. They include *P. aeruginosa* [34], *E. coli* [35], and *Staphylococci* [36]. Of further clinical importance is the fact that the organisms recovered here have also been implicated in the pathogenesis of other non-ocular infections such as diarrhea illnesses, urinary tract infections, sepsis, bacteremia, pneumonia, and meningitis [37–39]. In these infections, resistance rates higher than what we found for the least and most active anti-conjunctival antibiotics in this study were reported [38, 39]. Therefore, our antibiogram results indicate that the conjunctivitis cases studied bacteriologically are probably exogenous in origin. This possibility is further supported by the fact that all the patients studied are outpatients ruling out nosocomial ocular infections. The prevalence rate of plasmids of 44.6% can be said to be high, and this is synonymous with pathogens that elicit resistance to two or more antibiotics. Lower plasmid prevalence rates have been reported for pathogens such as *P. aeruginosa* [40], while higher rates have been reported for multidrug-resistant strains of *E. coli* and *K. pneumoniae* in epidemiological studies [41]. In this study, plasmids having a size range of 5.0–12.5 kb were common among the Gram-negative pathogens and those of 2.7–4.1 kb were common among the Gram-positive pathogens irrespective of the species affiliations, suggesting genetic diversity disparity but yet active transfer of plasmids and associated antibiotic resistances among the ocular pathogens in causing polymicrobial infections in patients with conjunctivitis in Lagos.

The implications of the results obtained from this study are twofold. First, the epidemiological situation of conjunctivitis is more complex than previously thought in this environment. Secondly, the etiologic agents harboring plasmids have the potentials of disseminating antibiotic resistance and their associated plasmids to other causative agents without plasmids that were previously sensitive to the commonly used anti-conjunctival agents in Lagos. To avert these implications, there is a need to carry out regular surveillance of agents of conjunctivitis in Lagos for antibiotic susceptibility and plasmid carriage in order to develop an appropriate strategy to prevent the spread of antibiotic resistance among pathogens and control conjunctivitis. Further studies are required using, apart from plasmid curing, techniques such as polymerase chain reaction and drug resistance phenotyping methods for better understanding of mechanisms to antibiotic resistance by Gram-positive and Gram-negative pathogens responsible for conjunctivitis and other ocular infections in Nigeria. Studies are also needed to understand the pathogenic disposition of some of these isolates, vis-a-vis the virulent factors they express using both in vitro and in vivo model systems. This is because the conjunctiva and cornea sac for instance naturally accommodate avirulent microbes as flora, and these organisms can be co-recovered from clinical eye specimens in patients with eye problems [9, 26].

Nevertheless, based on the findings of this study, it can be concluded that conjunctivitis as an eye problem in Lagos is polymicrobial with infections eliciting low and high sensitivity to chloramphenicol and ofloxacin, respectively. Infections are also associated with transferable R plasmids for chloramphenicol, ampicillin, and streptomycin. Continuous surveillance of conjunctivitis in relation to etiology, drug susceptibility, plasmid transferability, and mechanisms of resistance for epidemiological control in the study area is therefore recommended.
